# Spectrum and characterization of bi-allelic variants in *MMAB* causing *cblB*-type methylmalonic aciduria

**DOI:** 10.1007/s00439-021-02398-6

**Published:** 2021-11-18

**Authors:** Patrick Forny, Tanja Plessl, Caroline Frei, Celine Bürer, D. Sean Froese, Matthias R. Baumgartner

**Affiliations:** Division of Metabolism and Children’s Research Center, University Children’s Hospital Zurich, University of Zurich, 8032 Zurich, Switzerland

## Abstract

Pathogenic variants in *MMAB* cause *cblB*-type methylmalonic aciduria, an autosomal-recessive disorder of propionate metabolism. *MMAB* encodes ATP:cobalamin adenosyltransferase, using ATP and cob(I)alamin to create 5’-deoxyadenosylcobalamin (AdoCbl), the cofactor of methylmalonyl-CoA mutase (MMUT). We identified bi-allelic disease-causing variants in *MMAB* in 97 individuals with *cblB*-type methylmalonic aciduria, including 33 different and 16 novel variants. Missense changes accounted for the most frequent pathogenic alleles (p.(Arg186Trp), *N* = 57; p.(Arg191Trp), *N* = 19); while c.700C > T (p.(Arg234*)) was the most frequently identified truncating variant (*N* = 14). In fibroblasts from 76 affected individuals, the ratio of propionate incorporation in the presence and absence of hydroxocobalamin (PI ratio) was associated to clinical cobalamin responsiveness and later disease onset. We found p.(Arg234*) to be associated with cobalamin responsiveness in vitro, and clinically with later onset; p.(Arg186Trp) and p.(Arg191Trp) showed no clear cobalamin responsiveness and early onset. Mapping these and novel variants onto the MMAB structure revealed their potential to affect ATP and AdoCbl binding. Follow-up biochemical characterization of recombinant MMAB identified its three active sites to be equivalent for ATP binding, determined by fluorescence spectroscopy (*K*_d_ = 21 µM) and isothermal calorimetry (*K*_d_ = 14 µM), but function as two non-equivalent AdoCbl binding sites (*K*_d1_ = 0.55 μM; *K*_d2_ = 8.4 μM). Ejection of AdoCbl was activated by ATP (*K*_a_ = 24 µM), which was sensitized by the presence of MMUT (*K*_a_ = 13 µM). This study expands the landscape of pathogenic *MMAB* variants, provides association of in vitro and clinical responsiveness, and facilitates insight into MMAB function, enabling better disease understanding.

**Supplementary Information:**

The online version contains supplementary material available at 10.1007/s00439-021-02398-6.

## Introduction

The human ATP:cob(I)alamin adenosyltransferase enzyme (MMAB; EC 2.5.1.17) is a ubiquitously expressed protein involved in intracellular cobalamin (vitamin B_12_) metabolism. Its role involves the adenosylation of cob(I)alamin by ATP, creating adenosylcobalamin (AdoCbl), the cofactor of methylmalonyl-CoA mutase (MMUT; EC 5.4.99.2). This latter enzyme catalyzes the anaplerotic conversion of methylmalonyl-CoA to succinyl-CoA as part of the catabolism of valine, isoleucine, methionine, and threonine, as well as of odd-chain fatty acids and the side chain of cholesterol through the propionate pathway.

Bi-allelic pathogenic variants in the *MMAB* gene (OMIM #607,568) cause isolated methylmalonic aciduria of the *cblB*-type (MMA; OMIM #251,110). This disease can present with an early-onset severe form (presentation during the first month of life), whose symptoms include severe metabolic ketoacidosis, caused by accumulating acids (including methylmalonic acid) as well as lethargy potentially deteriorating to coma, which is partly owed to raised plasma ammonia levels (Forny et al. 2021). Patients with a later-onset typically present with a milder disease phenotype but are still likely to suffer from long-term complications which mainly affect the brain (neurological impairment) and kidney (renal insufficiency) (Hörster et al. 2007). Confirmation of diagnosis of *cblB*-type MMA classically involves assessment of residual propionate pathway function in patient fibroblasts by measuring propionate incorporation activity (PI activity) – a diagnostic assay which uses radio-labelled ^14^C-propionate as substrate and measures the incorporation of ^14^C atoms into acid precipitable proteins (Fowler et al. 2008). This is often performed in the presence and absence of hydroxocobalamin (OHCbl) in order to ascertain in vitro cobalamin responsiveness, reflected by the increased ratio of ^14^C-incorporation in the presence of OHCbl (Forny et al. 2016). Clinical response to OHCbl has not been systematically reported for *cblB* patients, but there is the potential that at least some patients whose fibroblasts show in vitro responsiveness may also benefit clinically from intramuscular cobalamin injections.

It was nearly 20 years ago that pathogenic variants in the *MMAB* gene, mapped to chromosome 12q24 and consisting of 9 exons, were found to be responsible for *cblB*-type MMA (Dobson et al. 2002). Today, 46 different disease associated variants have been described in *MMAB* according to the *Human Gene Mutation Database* (HGMD Professional version 2020.4), while 48 variants are classified as pathogenic or likely pathogenic on the ClinVar platform (https://www.ncbi.nlm.nih.gov/clinvar/, accessed 19 May 2021). Some of these variants are recurrent and have been characterized previously, particularly those arising on exon 7, which is considered to be a hotspot (Lerner-Ellis et al. 2006). The most frequently identified pathogenic variant to date is p.(Arg186Trp), which results in absent protein as assessed by Western blot analysis (Zhang et al. 2006). Another well characterized variant on exon 7 is p.(Arg191Trp), which has been shown to result in impaired ATP and cobalamin binding (Zhang et al. 2006) as well as thermal instability (Jorge‐Finnigan et al. 2010). Additionally, a frequently identified nonsense variant leading to the protein change p.(Gln234*) has been shown to result in high residual PI activity and OHCbl responsiveness in vitro (Lerner-Ellis et al. 2006).

Extensive biochemical characterization of bacterial PduO-type adenosyltransferases, a class to which MMAB also belongs, has illustrated their unique reaction mechanism (Johnson et al. 2004; Mera et al. 2007; Padovani et al. 2008; St. Maurice et al. 2008; Padovani and Banerjee 2009; Park et al. 2012; Mascarenhas et al. 2020). These studies have demonstrated that ATP binds to one of three potential active-sites, each occurring at the homotrimer subunit interfaces, following which cobalamin binds to the same site in the base-off state. The co-occurrence of ATP and cobalamin initiates nucleophilic attack of the C-5’ ribosyl-carbon of ATP, displacing triphosphate and forming AdoCbl, which is subsequently released following ATP binding to an adjacent active-site. Previous (Schubert and Hill 2006) and more recent (Campanello et al. 2018) structural and biochemical analyses of human MMAB have confirmed conservation of this overall architecture and mechanism, whereby each of MMAB’s three subunits are composed of a five-helix bundle with active sites located at the subunit interfaces. Nevertheless, as has been documented for other enzymes in the AdoCbl synthesis pathway (e.g., MMAA and MMUT (Froese et al. 2010; Plessl et al. 2017)), subtle alterations in oligomeric assembly between human and bacterial enzymes may have important consequences for their function and interaction, especially in the context of disease. Therefore, a thorough biochemical analysis of MMAB remains warranted.

In this study, we performed molecular genetics on 97 individuals with *cblB*-type MMA and examined PI activity in 76 of them. We report 16 novel disease-causing nucleotide changes in the *MMAB* gene and provide clear evidence that patients who are clinically responsive to OHCbl treatment display an equal responsiveness when their fibroblasts are assessed by the PI activity assay. We further show data indicating that biochemical parameters of MMAB, including ATP and AdoCbl affinity, differ from those of its structural homologs, which may influence the functional interaction of MMAB with downstream effectors such as MMUT and MMAA.

## Materials and methods

### Cohort of affected individuals and their samples

Primary fibroblast samples and corresponding patient information, including clinical and biochemical data, was collected from the years 1987 to 2021. The information and materials were obtained and used under the ethics approval no. KEK-2014–0211, amendment: PB_2020-00,053, granted by the Ethics Committee of the Canton of Zurich, Switzerland. Upon collection of the primary fibroblast cells, they were cultured under standard conditions, using Dulbecco’s modified Eagle medium (Gibco, Life Technologies, Zug, Switzerland) with 10% fetal bovine serum (Gibco) and antibiotics (GE Healthcare, Little Chalfont, UK). All individuals were diagnosed with *cblB*-type MMA either through complementation analysis, propionate incorporation studies, or molecular genetic testing of the *MMAB* gene.

### Propionate incorporation assay

Propionate incorporation into acid precipitable material of primary fibroblasts was assessed according to a protocol described previously (Willard et al. 1976) with modification as described (Froese and Baumgartner 2020). The assay was performed without and with supplemented OHCbl at 1 μg/mL to calculate the propionate incorporation ratio (PI ratio; PI activity with OHCbl divided by PI activity without OHCbl).

### Genotyping

Cultivated fibroblasts or frozen fibroblast cell pellets which were previously cultivated were directly used for genomic DNA extraction with the DNeasy Blood & Tissue Kit (Cat. No./ID: 69,506; Qiagen) following manufacturer’s instructions. Amplification of *MMAB* exons and flanking intronic sequences was performed using forward and reverse primers described in Suppl. Table 1. Sequencing was performed according to the Sanger sequencing principle by using the BigDye Terminator v1 Cycle Sequencing Kit (Thermo Fisher Scientific) according to manufacturer’s instructions. *MMAB* variants are described according to HGVS nomenclature version 2.0 as verified with the Mutalyzer software (version 2.0.34, 15 March 2021, accessed 11 May 2021 on https://mutalyzer.nl) based on the NCBI Reference Sequence NG_007096.1. Novel variants have been submitted to NCBI ClinVar (https://www.ncbi.nlm.nih.gov/clinvar/). All novel variants were found in either compound heterozygous or homozygous state in fibroblasts which showed diminished propionate incorporation activity, providing functional evidence validating their pathogenicity. The homozygous variant c.87 T > A in patient ID 32 resulting in the predicted early truncation p.(Tyr29*) showed confirmed inheritance of one allele from the mother. The known variant c.291-G > A (same nucleotide position is affected in the novel variant c.291-1G > T) was found in patients 57 and 61 heterozygous with known disease-causing variants, whose fibroblasts show impaired propionate incorporation.

### Cloning, expression, and purification of recombinant proteins

Recombinant human MMAB and MMUT were produced as previously described (Plessl et al. 2017). Purified proteins were exchanged into Buffer A (100 mM HEPES pH 7.5, 300 mM KCl, 250 µM MgCl_2_, 5% glycerol) which was used for all subsequent experiments.

### UV–visible spectroscopy

AdoCbl binding and release was monitored by spectrophotometry, as previously described (Plessl et al. 2017). Briefly, for AdoCbl-binding, MMAB or MMUT in Buffer A were added to 35 μM AdoCbl and spectra from 300 to 700 nm recorded immediately after mixing. For release of AdoCbl, ATP (0–1650 μM) was added to holo-MMAB or holo-MMUT (60 μM) in the absence or presence of the other proteins (60 μM) and spectra from 300 to 700 nm recorded. To determine *K*_d_ or *K*_a_, the change in fluorescence at 525 nm was compared to concentration of substrate.

### ATP binding assays

Binding of the fluorescent ATP analogue 2',3'-O-(N-Methyl-anthraniloyl)-adenosine-5'-triphosphate (MANT-ATP) was measured by titration of MMAB (0–55 µM) to 10 µM MANT-ATP in Buffer A. Binding was estimated by plotting change in fluorescence at 444 nm (excitation: 360 nm) monitored by a Synergy HT plate reader. Isothermal calorimetry was performed using a MicroCal VP-ITC with a sample cell volume of 1.8 ml and 300 µl injection syringe at 20 °C. 28 × 10 µl injections of 5 mM ATP were made to 200 µM MMAB. Isothermal calorimetry data were analyzed using MicroCal Origin (v7.0) software.

### Size-exclusion chromatography

Analytical gel filtration was performed on a Superose 6 Increase 10/300 GL column (GE Healthcare) pre-equilibrated with Buffer A. The column was calibrated using carbonic anhydrase (29 kDa), alcohol dehydrogenase (150 kDa) and apo-ferritin (443 kDa) (Sigma-Aldrich) as molecular-weight standards. Unless otherwise indicated, 100 µl of each protein was injected at a concentration of 25 µM. Where indicated, fractions were collected and examined by 4–20% Tris–Glycine SDS-PAGE (ThermoFisher Scientific) and stained using InstantBlue coomassie Stain (Expedeon) or by Western blot analysis following transfer to a Protran BA85 nitrocellulose membrane (Whatman), blocking for 1 h with 0.5% skim milk, and visualized with anti-MMAB (1:1000, HPA039017 Sigma-Aldrich), anti-mouse HRP (1:5000, ab131368 Abcam) and ECL Western blotting Detection Reagent (GE Healthcare).

### Software

R software version 4.0.4 was used to design figures and calculate significance levels. Curve fitting for binding of ligands was performed using the (one site specific binding) model *Y* = *B*_max_*X/(K_*d*_ + *X)* (B_max_, maximum specific binding; *K*_d_: equilibrium dissociation constant). For the dissociation curve, model fitting using the formula *Y* = (Y0-NS)*exp(-K*X) + *NS* (Y0, binding at lowest concentration; NS, nonspecific binding at infinite concentration; K, rate constant) was performed. Statistical tests for significance are indicated in figure legend. The structure figure was designed with Molsoft ICM software, using PDB ID 6D5K as a basis. Schematic depiction of AdoCbl chemical states was created with BioRender.com.

## Results

### Identification of pathogenic *MMAB* variants

**Fig. 1:**
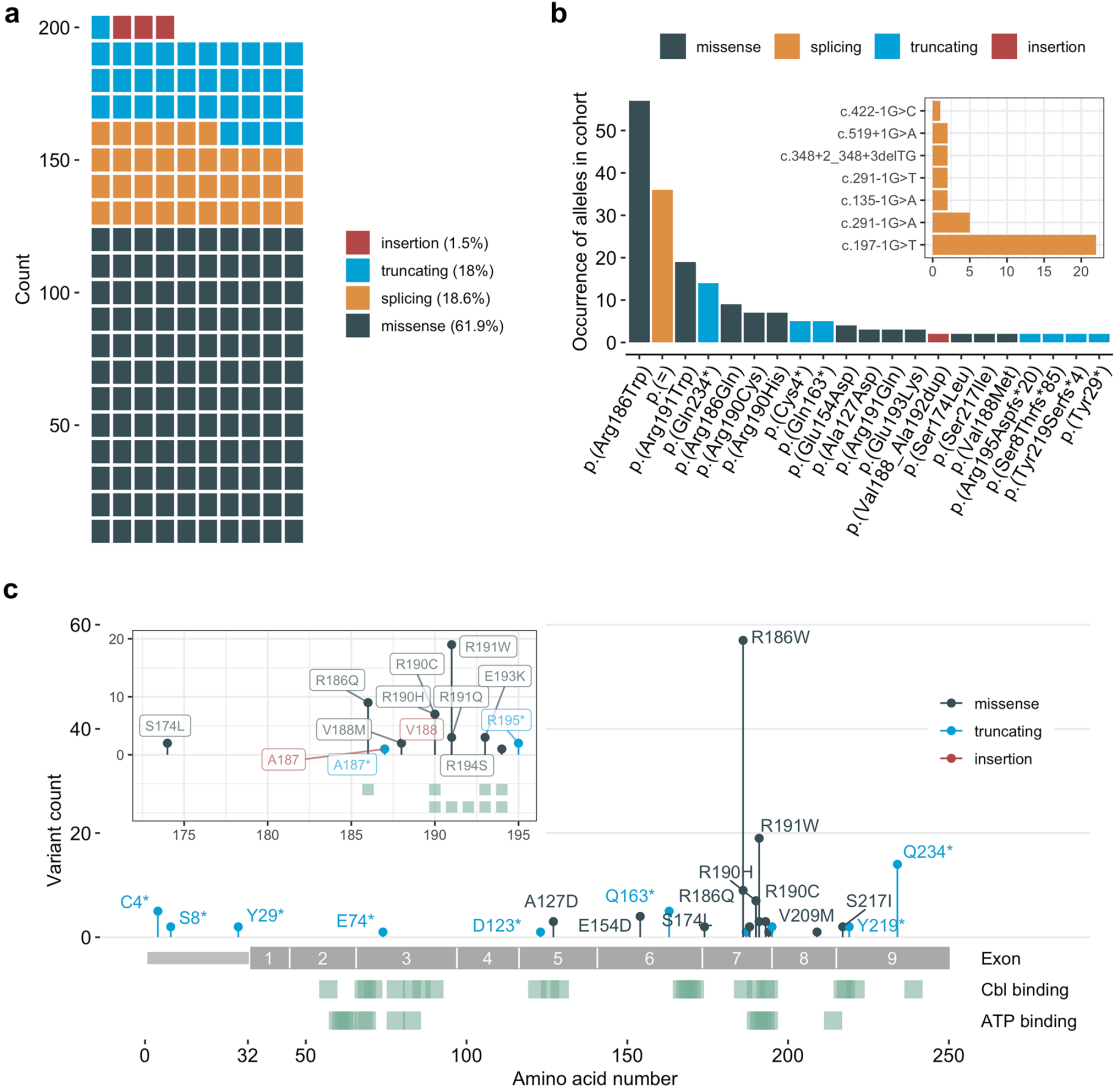
Distribution and quantification of pathogenic *MMAB* variants. (**a**) Count of different variant types in our cohort; each square corresponds to one identified allele. (**b**) Ranked list of variants, which occurred at least twice in the cohort, from highest to lowest frequency (inset: ranked list of splicing variants). (**c**) Lolliplot of all missense, truncating and insertion variants, distributed along the MMAB polypeptide chain (inset: zoom of the hotspot region at the end of exon 7). Tracks underneath the polypeptide chain indicate residues involved in cobalamin and ATP binding

This study presents information obtained from 97 individuals with confirmed *cblB*-type MMA, all of which were found to carry two disease-causing variants in the *MMAB* gene. A summary of the affected individuals and the identified variants are presented in Table 1, 2, respectively. In total we identified 33 different variants, of which 16 were novel, including the predicted missense variants c.380C > A (p.(Ala127Asp)), c.462G > T (p.(Glu154Asp)) and c.650G > T (p.(Ser217Ile)). Variants resulting in single amino acid substitutions accounted for the majority of identified changes (61.9%), while splicing and truncating changes were the next most frequent, constituting 18.6% and 18% of variants, respectively (Fig. 1a). Some changes occurred at a high frequency in the cohort (Table 2). These included the missense alleles p.(Arg186Trp) and p.(Arg191Trp) (57 and 19 alleles, respectively), and the splicing variant c.197-1G > T (22 alleles) (Fig. 1b). The most frequently identified truncating variant was c.700C > T (14 alleles), which results in a predicted premature termination of translation at peptide position 234 (p.(Gln234*)) (Fig. 1b). The majority of disease-causing variants identified resulted in predicted changes to the C-terminal half of the polypeptide chain (Fig. 1c). A particular hotspot was identified in exon 7, corresponding to amino acids 173–195, which contribute important residues to the binding sites of cobalamin and ATP (Fig. 1c and inset). These changes are likely to result in a direct impact on catalytic activity.

**Table 1:** Overview of all 97 patients included in the study cohort in increasing order of the nucleotide allele 1, followed by order of allele 2

ID	Allele 1		Allele 2		PI activity -OHCbl	PI activity + OHCbl	PI ratio	Clinical B_12_ response	Age at onset [days]
	Nucleotide change	Amino acid change	Nucleotide change	Amino acid change					
111	c.12C > A	p.(Cys4*)	c.12C > A	p.(Cys4*)	1252	1840	1.47	No	2555
112	c.12C > A	p.(Cys4*)	c.12C > A	p.(Cys4*)	195	197	1.01	No	548
29	c.23delG	p.(Ser8Thrfs*85)	c.23delG	p.(Ser8Thrfs*85)	412	418	1.01	–	–
32	c.87 T > A	p.(Tyr29*)	c.87 T > A	p.(Tyr29*)	–	–	–	–	–
86	c.135-1G > A	p.( =)	c.556C > T	p.(Arg186Trp)	–	–	–	–	–
24	c.197-1G > T	p.( =)	c.197-1G > T	p.( =)	–	–	–	–	–
31	c.197-1G > T	p.( =)	c.197-1G > T	p.( =)	1420	1430	1.01	–	–
64	c.197-1G > T	p.( =)	c.197-1G > T	p.( =)	–	–	–	–	–
72	c.197-1G > T	p.( =)	c.197-1G > T	p.( =)	561	709	1.26	–	–
82	c.197-1G > T	p.( =)	c.197-1G > T	p.( =)	659	795	1.21	–	90
132	c.197-1G > T	p.( =)	c.197-1G > T	p.( =)	–	–	-	–	–
95	c.197-1G > T	p.( =)	c.197-1G > T	p.( =)	506	471	0.93	–	–
131	c.197-1G > T	p.( =)	c.197-1G > T	p.( =)	–	–	-	–	–
123	c.197-1G > T	p.( =)	c.197-1G > T	p.( =)	413	377	0.91	No	4
127	c.197-1G > T	p.( =)	c.197-1G > T	p.( =)	860	940	1.09	–	3
39	c.197-1G > T	p.( =)	c.556C > T	p.(Arg186Trp)	354	901	2.55	Yes	1000
33	c.220G > T	p.(Glu74*)	c.556C > T	p.(Arg186Trp)	1970	1850	0.94	–	–
129	c.291-1G > A	p.( =)	c.291-1G > A	p.( =)	–	–	–	–	–
57	c.291-1G > A	p.( =)	c.572G > A	p.(Arg191Gln)	493	1511	3.07	-	-
61	c.291-1G > A	p.( =)	c.625G > A	p.(Val209Met)	5083	10,116	1.99	Yes	1000
25	c.291-1G > T	p.( =)	c.291-1G > T	p.( =)	–	–	–	–	–
34	c.348 + 2_348 + 3delTG	p.( =)	c.348 + 2_348 + 3delTG	p.( =)	379	334	0.88	-	5
88	c.367del	p.(Asp123Thrfs*18)	c.556C > T	p.(Arg186Trp)	1210	1050	0.87	No	3
130	c.380C > A	p.(Ala127Asp)	c.197-1G > T	p.( =)	–	–	–	–	–
97	c.380C > A	p.(Ala127Asp)	c.380C > A	p.(Ala127Asp)	2876	6231	2.17	Yes	1095
103	c.422-1G > C	p.( =)	c.568C > T	p.(Arg190Cys)	3362	4228	1.26	Yes	180
115	c.462G > T	p.(Glu154Asp)	c.462G > T	p.(Glu154Asp)	1280	1244	0.97	–	2
116	c.462G > T	p.(Glu154Asp)	c.462G > T	p.(Glu154Asp)	–	–	–	–	–
26	c.487C > T	p.(Gln163*)	c.487C > T	p.(Gln163*)	561	608	1.08	–	–
94	c.487C > T	p.(Gln163*)	c.487C > T	p.(Gln163*)	860	960	1.12	–	–
63	c.487C > T	p.(Gln163*)	c.562G > A	p.(Val188Met)	4100	6200	1.51	–	1000
105	c.519 + 1G > A	p.( =)	c.519 + 1G > A	p.( =)	1930	2030	1.05	–	–
114	c.521C > T	p.(Ser174Leu)	c.521C > T	p.(Ser174Leu)	–	–	–	–	–
65	c.556C > T	p.(Arg186Trp)	c.12C > A	p.(Cys4*)	1120	3547	3.17	–	–
21	c.556C > T	p.(Arg186Trp)	c.556C > T	p.(Arg186Trp)	601	800	1.33	No	4
128	c.556C > T	p.(Arg186Trp)	c.556C > T	p.(Arg186Trp)	–	–	–	–	–
36	c.556C > T	p.(Arg186Trp)	c.556C > T	p.(Arg186Trp)	566	868	1.53	–	–
42	c.556C > T	p.(Arg186Trp)	c.556C > T	p.(Arg186Trp)	580	790	1.36	–	–
49	c.556C > T	p.(Arg186Trp)	c.556C > T	p.(Arg186Trp)	530	490	0.92	Yes	5
51	c.556C > T	p.(Arg186Trp)	c.556C > T	p.(Arg186Trp)	–	–	–	Yes	135
55	c.556C > T	p.(Arg186Trp)	c.556C > T	p.(Arg186Trp)	638	682	1.07	–	–
62	c.556C > T	p.(Arg186Trp)	c.556C > T	p.(Arg186Trp)	–	–	–	–	–
68	c.556C > T	p.(Arg186Trp)	c.556C > T	p.(Arg186Trp)	1340	2060	1.54	–	–
69	c.556C > T	p.(Arg186Trp)	c.556C > T	p.(Arg186Trp)	700	1090	1.56	–	–
70	c.556C > T	p.(Arg186Trp)	c.556C > T	p.(Arg186Trp)	1026	1477	1.44	Yes	3
81	c.556C > T	p.(Arg186Trp)	c.556C > T	p.(Arg186Trp)	430	700	1.63	–	–
102	c.556C > T	p.(Arg186Trp)	c.556C > T	p.(Arg186Trp)	825	1176	1.42	–	–
106	c.556C > T	p.(Arg186Trp)	c.556C > T	p.(Arg186Trp)	457	640	1.4	No	16
119	c.556C > T	p.(Arg186Trp)	c.556C > T	p.(Arg186Trp)	753	954	1.27	–	–
120	c.556C > T	p.(Arg186Trp)	c.556C > T	p.(Arg186Trp)	561	680	1.21	–	–
121	c.556C > T	p.(Arg186Trp)	c.556C > T	p.(Arg186Trp)	–	–	–	–	–
125	c.556C > T	p.(Arg186Trp)	c.556C > T	p.(Arg186Trp)	527	-	-	No	120
28	c.556C > T	p.(Arg186Trp)	c.557G > A	p.(Arg186Gln)	220	270	1.23	–	4
47	c.556C > T	p.(Arg186Trp)	c.557G > A	p.(Arg186Gln)	–	–	–	No	3
98	c.556C > T	p.(Arg186Trp)	c.557G > A	p.(Arg186Gln)	594	472	0.79	–	–
101	c.556C > T	p.(Arg186Trp)	c.557G > A	p.(Arg186Gln)	2516	5737	2.28	Yes	455
133	c.556C > T	p.(Arg186Trp)	c.557G > A	p.(Arg186Gln)	–	–	–	–	–
41	c.556C > T	p.(Arg186Trp)	c.558_559delinsC	p.(Ala187Profs*27)	339	323	0.95	No	2
85	c.556C > T	p.(Arg186Trp)	c.563_577dup	p.(Val188_Ala192dup)	2170	2480	1.14	–	–
17	c.556C > T	p.(Arg186Trp)	c.563_577dup	p.(Val188_Ala192dup)	1600	4540	2.84	–	210
18	c.556C > T	p.(Arg186Trp)	c.568C > T	p.(Arg190Cys)	1232	2368	1.92	–	300
122	c.556C > T	p.(Arg186Trp)	c.571C > T	p.(Arg191Trp)	1060	1480	1.4	No	-
37	c.556C > T	p.(Arg186Trp)	c.700C > T	p.(Gln234*)	1716	3267	1.9	Yes	1000
38	c.556C > T	p.(Arg186Trp)	c.700C > T	p.(Gln234*)	1353	2262	1.67	Yes	90
46	c.556C > T	p.(Arg186Trp)	c.700C > T	p.(Gln234*)	–	–	–	–	–
53	c.556C > T	p.(Arg186Trp)	c.700C > T	p.(Gln234*)	862	3764	4.37	Yes	820
77	c.556C > T	p.(Arg186Trp)	c.700C > T	p.(Gln234*)	1780	9870	5.54	No	300
117	c.556C > T	p.(Arg186Trp)	c.700C > T	p.(Gln234*)	2755	9200	3.34	–	–
59	c.557G > A	p.(Arg186Gln)	c.557G > A	p.(Arg186Gln)	346	376	1.09	–	–
76	c.557G > A	p.(Arg186Gln)	c.557G > A	p.(Arg186Gln)	–	–	–	–	–
66	c.560_561insGGCACGGGC	p.(Ala187_Val188insAlaArgAla)	c.135-1G > A	p.( =)	870	830	0.95	No	-
40	c.562G > A	p.(Val188Met)	c.582A > T	p.(Arg194Ser)	1978	4820	2.44	Yes	10
44	c.568C > T	p.(Arg190Cys)	c.568C > T	p.(Arg190Cys)	–	–	–	–	–
43	c.568C > T	p.(Arg190Cys)	c.568C > T	p.(Arg190Cys)	432	435	1.01	–	–
80	c.568C > T	p.(Arg190Cys)	c.571C > T	p.(Arg191Trp)	378	393	1.04	Yes	2
71	c.569G > A	p.(Arg190His)	c.569G > A	p.(Arg190His)	565	668	1.18	–	–
75	c.569G > A	p.(Arg190His)	c.569G > A	p.(Arg190His)	540	1770	3.28	–	180
15	c.569G > A	p.(Arg190His)	c.569G > A	p.(Arg190His)	1850	6020	3.25	–	–
52	c.569G > A	p.(Arg190His)	c.571C > T	p.(Arg191Trp)	730	1920	2.63	–	–
22	c.571C > T	p.(Arg191Trp)	c.571C > T	p.(Arg191Trp)	–	–	–	–	15
45	c.571C > T	p.(Arg191Trp)	c.571C > T	p.(Arg191Trp)	880	1069	1.21	–	–
48	c.571C > T	p.(Arg191Trp)	c.571C > T	p.(Arg191Trp)	1175	1428	1.21	No	5
74	c.571C > T	p.(Arg191Trp)	c.571C > T	p.(Arg191Trp)	1410	1370	0.97	–	–
79	c.571C > T	p.(Arg191Trp)	c.571C > T	p.(Arg191Trp)	826	5838	7.07	No	240
93	c.571C > T	p.(Arg191Trp)	c.571C > T	p.(Arg191Trp)	630	640	1.02	–	3
104	c.571C > T	p.(Arg191Trp)	c.571C > T	p.(Arg191Trp)	1000	960	0.96	–	-
126	c.571C > T	p.(Arg191Trp)	c.571C > T	p.(Arg191Trp)	1040	–	–	–	–
118	c.572G > A	p.(Arg191Gln)	c.572G > A	p.(Arg191Gln)	710	1980	2.79	Yes	3
87	c.577G > A	p.(Glu193Lys)	c.577G > A	p.(Glu193Lys)	478	455	0.95	–	–
78	c.577G > A	p.(Glu193Lys)	c.700C > T	p.(Gln234*)	790	4800	6.08	–	–
109	c.581_582dup	p.(Arg195Aspfs*20)	c.581_582dup	p.(Arg195Aspfs*20)	570	610	1.07	–	–
27	c.650G > T	p.(Ser217Ile)	c.650G > T	p.(Ser217Ile)	866	825	0.95	No	2
73	c.656_659del	p.(Tyr219Serfs*4)	c.656_659del	p.(Tyr219Serfs*4)	1318	6630	5.03	No	240
54	c.700C > T	p.(Gln234*)	c.291-1G > A	p.( =)	599	2324	3.88	Yes	3
58	c.700C > T	p.(Gln234*)	c.700C > T	p.(Gln234*)	1026	4848	4.73	Yes	2
11	c.700C > T	p.(Gln234*)	c.700C > T	p.(Gln234*)	780	3760	4.82	Yes	180
124	c.700C > T	p.(Gln234*)	c.700C > T	p.(Gln234*)	910	3200	3.52	Yes	150

Unit of PI activity is nmol ^14^C/mg protein/16 h. Dashes (“ − “) indicate information, which were not available

**Table 2:** List of variants as detected in the study cohort in increasing order of the nucleotide allele

Nucleotide change	Exon/intron	Predicted amino acid change	Allele type	Frequency in cohort	Accession no. (ClinVar)	Reference
c.12C > A	Exon 1	p.(Cys4*)	Truncating	5	SCV000789791.1	(Illson et al. 2013)
c.23delG	Exon 1	p.(Ser8Thrfs*85)	Truncating	2	Submitted to ClinVar	This study
c.87 T > A	Exon 1	p.(Tyr29*)	Truncating	2	Submitted to ClinVar	This study
c.135-1G > A	Intron 1	p.( =)	Splicing	2	Submitted to ClinVar	This study
c.197-1G > T	Intron 2	p.( =)	Splicing	22	SCV000699779.1	(Dobson et al. 2002)
c.220G > T	Exon 3	p.(Glu74*)	Truncating	1	Submitted to ClinVar	This study
c.291-1G > A	Intron 3	p.( =)	Splicing	5	SCV000023400.1	(Dobson et al. 2002)
c.291-1G > T	Intron 3	p.( =)	Splicing	2	Submitted to ClinVar	This study
c.348 + 2_348 + 3delTG	Exon 4	p.( =)	Splicing	2	Submitted to ClinVar	This study
c.367del	Exon 5	p.(Asp123Thrfs*18)	Truncating	1	Submitted to ClinVar	This study
c.380C > A	Exon 5	p.(Ala127Asp)	Missense	3	Submitted to ClinVar	This study
c.422-1G > C	Itron 5	p.( =)	Splicing	1	Submitted to ClinVar	This study
c.462G > T	Exon 6	p.(Glu154Asp)	Missense	4	Submitted to ClinVar	This study
c.487C > T	Exon 6	p.(Gln163*)	Truncating	5	Submitted to ClinVar	This study
c.519 + 1G > A	Intron 6	p.( =)	Splicing	2	SCV000938892.1	(Lerner-Ellis et al. 2006)
c.521C > T	Exon 7	p.(Ser174Leu)	Missense	2	SCV000610289.1	(Lerner-Ellis et al. 2006)
c.556C > T	Exon 7	p.(Arg186Trp)	Missense	57	SCV000023399.1	(Dobson et al. 2002)
c.557G > A	Exon 7	p.(Arg186Gln)	Missense	9	SCV000791878.1	(Dobson et al. 2002)
c.558_559delinsC	Exon 7	p.(Ala187Profs*27)	Truncating	1	Submitted to ClinVar	This study
c.560_561insGGCACGGGC	Exon 7	p.(Ala187_Val188insAlaArgAla)	Insertion	1	Submitted to ClinVar	This study
c.562G > A	Exon 7	p.(Val188Met)	Missense	2	SCV000790795.1	(Liu et al. 2015)
c.563_577dup	Exon 7	p.(Val188_Ala192dup)	Insertion	2	SCV000794600.1	(Lerner-Ellis et al. 2006)
c.568C > T	Exon 7	p.(Arg190Cys)	Missense	7	SCV001132248.1	(Lerner-Ellis et al. 2006)
c.569G > A	Exon 7	p.(Arg190His)	Missense	7	SCV000791445.1	(Lerner-Ellis et al. 2006)
c.571C > T	Exon 7	p.(Arg191Trp)	Missense	19	SCV000331820.4	(Dobson et al. 2002)
c.572G > A	Exon 7	p.(Arg191Gln)	Missense	3	SCV001218261.2	(Lerner-Ellis et al. 2006)
c.577G > A	Exon 7	p.(Glu193Lys)	Missense	3	SCV000794321.1	(Dobson et al. 2002)
c.581_582dup	Exon 7	p.(Arg195Aspfs*20)	Truncating	2	Submitted to ClinVar	This study
c.582A > T	Exon 7	p.(Arg194Ser)	Missense	1	SCV000493330.11	Not published
c.625G > A	Exon 8	p.(Val209Met)	Missense	1	SCV000336947.4	Not published
c.650G > T	Exon 9	p.(Ser217Ile)	Missense	2	Submitted to ClinVar	This study
c.656_659del	Exon 9	p.(Tyr219Serfs*4)	Truncating	2	Submitted to ClinVar	This study
c.700C > T	Exon 9	p.(Gln234*)	Truncating	14	SCV000245632.1	(Lerner-Ellis et al. 2006)

### Clinical and biochemical cohort characterization

In addition to molecular genetic information, we assessed enzymatic function, in the form of propionate incorporation (PI) activity, from fibroblasts of 76 individuals (Table 1). PI activity was performed in the absence and presence of hydroxocobalamin (OHCbl) supplementation to the assay media. On average, PI activity in media supplemented with OHCbl was higher than in its absence, but for each individual both measures correlated strongly (Table 1, Suppl. Figure 1a).

**Fig. 2:**
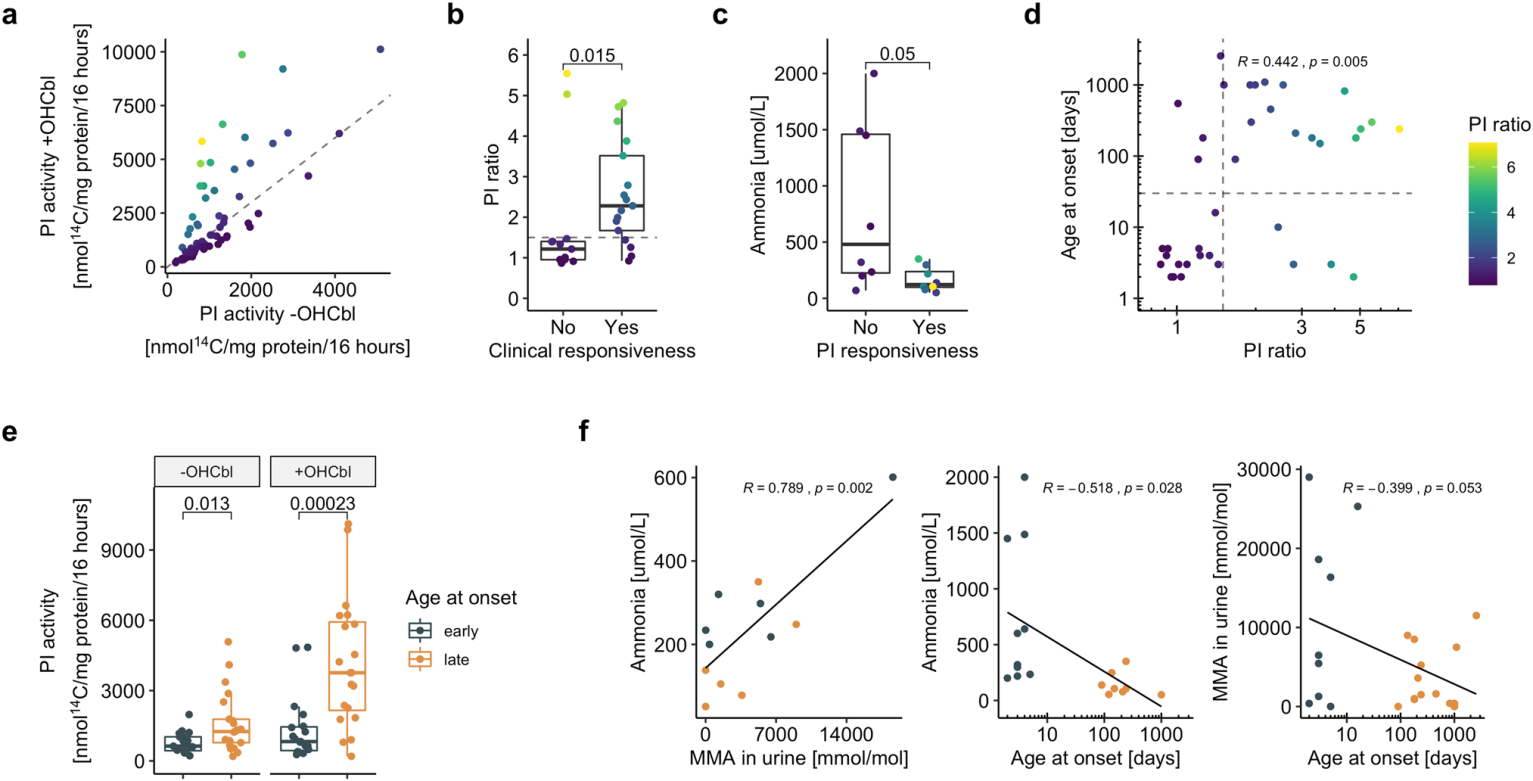
Clinical and biochemical cohort characterization. (**a**) Scatter plot of PI activity with and without supplementation of OHCbl; dashed line indicates a PI ratio of 1.5. (**b**) In vivo vitamin B_12_ response (clinical responsiveness) compared to in vitro responsiveness (PI ratio, dashed line indicates ratio at 1.5). (**c**) In vitro OHCbl response (PI responsiveness) compared to ammonia levels as assessed at time at presentation. (**d**) Scatter plot comparing PI ratio to age at onset; vertical dashed line indicates PI ratio at 1.5, horizontal dashed line indicates age at onset of 30 days. (**e**) PI activity with and without supplementation of OHCbl, grouped in early and late onset. (**f**) Linear regression plots comparing clinical and biochemical parameters. *p* values in (**b**), (**c**) and (**e**) are calculated by Wilcoxon signed-rank test. Linear regressions in (**d**) and (**f**) are calculated by Pearson correlation

The ratio of PI activity with and without OHCbl supplementation (PI ratio; Fig. 2a), typically using a cut-off of 1.5, has been used to discriminate between cells with residual activity that are responsive to OHCbl supplementation and those without (Fowler et al. 2008). Here, we found that individuals who were clinically responsive to vitamin B_12_ supplementation provided fibroblasts with a much higher PI ratio than those who were not, and the majority of clinically responsive individuals indeed had a PI ratio higher than 1.5 (Fig. 2b). Thus, in the context of MMAB deficiency, a PI ratio cut-off of 1.5 may be used to discriminate between cobalamin responsive and non-responsive fibroblasts. Accordingly, individuals whose fibroblasts were cobalamin responsive tended to have a lower maximal plasma ammonia at presentation (Fig. 2c). Finally, there was a clear positive correlation between PI ratio and the age at onset of presentation (Fig. 2d), which was further supported by the fact that individuals who presented with a later onset (> 30 days) had fibroblasts with a higher PI activity following OHCbl supplementation (Fig. 2e).

From a clinical perspective, there is an important interrelation between an early age at onset of presentation and the maximal measured concentrations of plasma ammonia and urinary methylmalonic acid, as all three parameters have been implicated in a worsened disease progression in MMA (Hörster et al. 2007; Kölker et al. 2015). For the individuals with available data in our cohort, we found that the latter two biochemical variables correlated positively with each other, while they were negatively correlated with age at onset (Fig. 2f), which showed two density peaks in our cohort at 2.5 and 300 days, respectively (Suppl. Figure 1b). These findings reinforce the predictive value of PI activity and the PI ratio, which are strongly correlated to these same clinical and biochemical parameters.

### Functional impact of variant classes

Since PI activity and PI ratio correlate with important clinical parameters, we sought to use them to investigate the genotype–phenotype correlation of variant types and the presence of specific (frequent) variants found in our cohort.

**Fig. 3:**
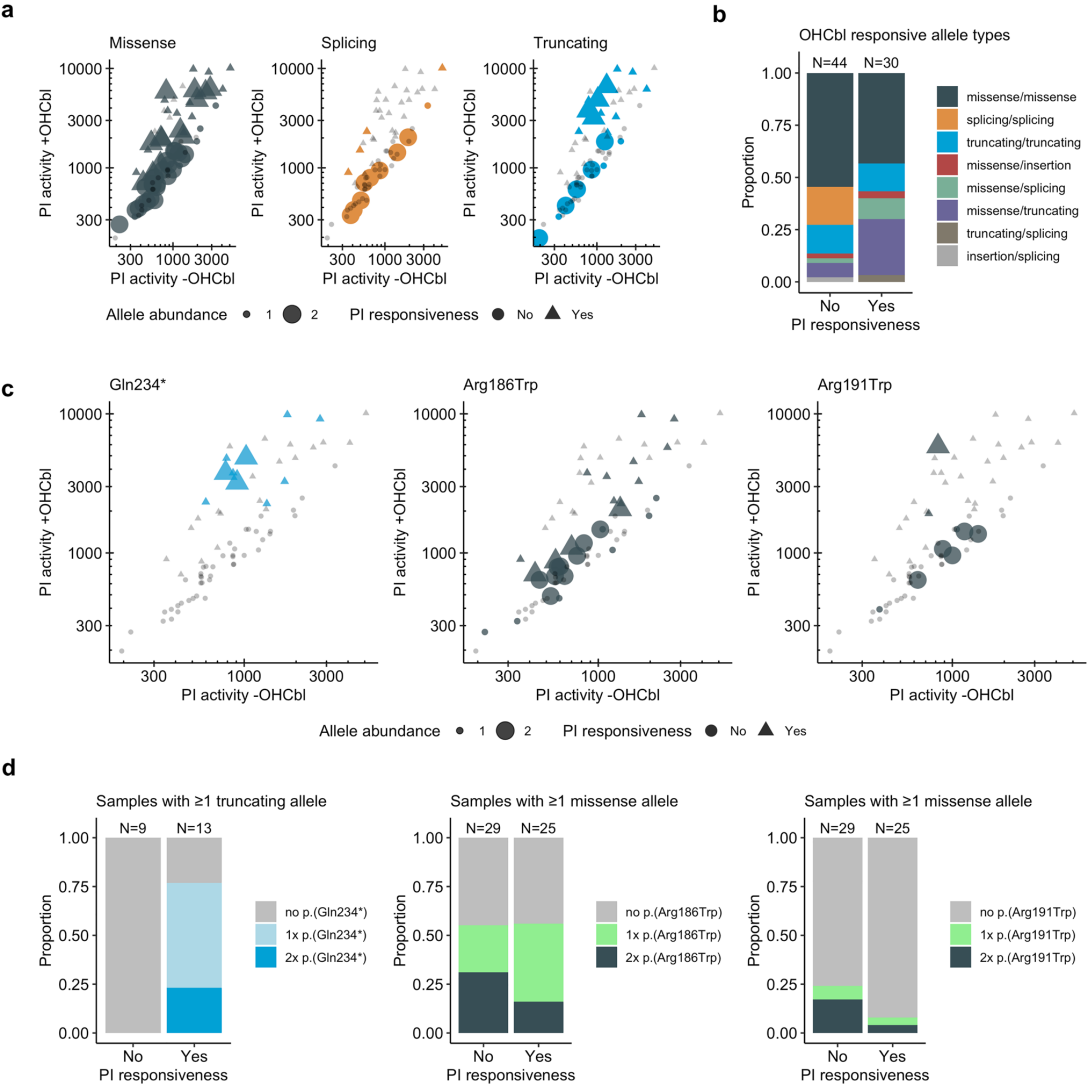
Functional impact of variant types and specific variants. (**a**) Scatter plot of PI activity with and without supplementation of OHCbl, grouped according to variant types indicated by colors; triangles indicate in vitro responsiveness, dot or triangle size indicate the abundance of the specific allele category. (**b**) Proportional abundance of different allele combinations, grouped according to in vitro responsiveness. (**c**) same as (**a**), titles indicate which specific alleles are color coded. (**d**) same as (**b**) but with color coded abundance of specific alleles

Examination of variant classes as a function of PI activity with and without OHCbl supplementation reveals missense and truncating variants to distribute across the entire range of very low to very high PI activity and present as both OHCbl-responsive and unresponsive (Fig. 3a). Since missense changes have the potential impact range of very disruptive to mild alterations, depending on the nature and location of the substitution, for them such a finding might be expected. However, finding very high PI activity and OHCbl-responsiveness as a consequence of premature truncation is more surprising. This latter, however, appears to be driven by a single variant, p.(Gln234*), as discussed further below. In contrast to missense and truncating variants, splicing variants almost exclusively resulted in low to moderate PI activity.

We further determined the contribution of each variant class to OHCbl responsiveness by plotting the proportion of responsive and non-responsive cells according to variant type (Fig. 3b). Consistent with their low PI activity in the presence and absence of OHCbl, cells harboring splicing variants on both alleles (splicing/splicing) were found only in the non-responsive category. By contrast, a larger proportion of cells with the combination of missense/truncating alleles were found to be responsive than non-responsive. Nevertheless, cells containing two missense variants (missense/missense) or two truncating variants (truncating/truncating) were found to be responsive with a relative chance of about 50%.

### Functional and clinical impact of frequent variants

We investigated the contribution of the most frequently identified alleles to PI activity (Fig. 3c) and responsiveness (Fig. 3d). For p.(Gln234*), the most frequently identified truncating variant (*N* = 14), cells that contained this variant in one or two alleles had medium to high residual activity and all were responsive to the presence of OHCbl. Correspondingly, 6/7 individuals harboring this variant, including all 3 in the homozygous state, from which we had clinical information, were described as clinically responsive to vitamin B_12_ (Table 1). Consistent with this dual responsiveness, two homozygous individuals showed a later onset (150 and 180 days) and one had onset at 2 days of age, while 4/5 individuals heterozygous for this variant showed a later onset (3, 90, 300, 820 days; one asymptomatic) (Table 1). The only other truncating variant associated with in vitro OHCbl responsiveness was p.(Tyr219Serfs*4), identified once in the homozygous state (Suppl. Figure 1c); all others were associated with non-responsiveness. Therefore, given its high frequency amongst our cohort, p.(Gln234*) was the source of the generally high PI activity and OHCbl responsiveness found in all cells with truncating variants and may be considered a cobalamin responsive variant.

By contrast, no clear dose-dependent allelic response was identified for the missense variant p.(Arg186Trp) (Fig. 3c, d), the most frequently identified allele in our cohort. It was detected in a homozygous state in fibroblasts from 18 individuals, which showed low PI activity and a median PI ratio of 1.4, corresponding to a weak or no response to OHCbl addition (Suppl. Figure 1d). Correspondingly, cell lines harboring this variant in one or two alleles were identified to be responsive approximately 50% of the time. Consistently, 7 of the 14 individuals containing this variant in one (3/7) or two (4/7) alleles were described to be clinically responsive to vitamin B_12_, while individuals carrying this variant in a homozygous state and of which information was available had mostly (4/5) early-onset of disease (Table 1). A different amino acid exchange at the same position (p.(Arg186Gln)) also resulted in non-responsiveness (Suppl. Figure 1e)*.*

Similarly, fibroblasts from individuals homozygous for p.(Arg191Trp), the second most frequently identified missense variant, showed moderate activity with no clear indication of cobalamin responsiveness (Fig. 3c, d). Individuals homozygous for this variant had mostly (3/4) early-onset of disease, while a heterozygous individual (with p.(Arg190Cys)) and an individual homozygous for p.(Arg191Gln) also had early onset disease (Table 1).

In regards to the novel missense variants identified in this study, p.(Ala127Asp) was identified once each in the homozygous and heterozygous state, showing in vitro and in vivo response to cobalamin in the homozygous state (Suppl. Figure 1e, Table 1). p.(Glu154Asp) was identified twice in the homozygous state, and was not cobalamin responsive (Suppl. Figure 1e), while we unfortunately received no information about their clinical responsiveness (Table 1). Finally, p.(Ser217Ile) was identified in one homozygous individual, whose cells displayed low PI activity without cobalamin responsiveness (Suppl. Figure 1e), and was clinically not responsive to cobalamin administration (Table 1).

### Molecular consequences of missense variants

**Fig. 4:**
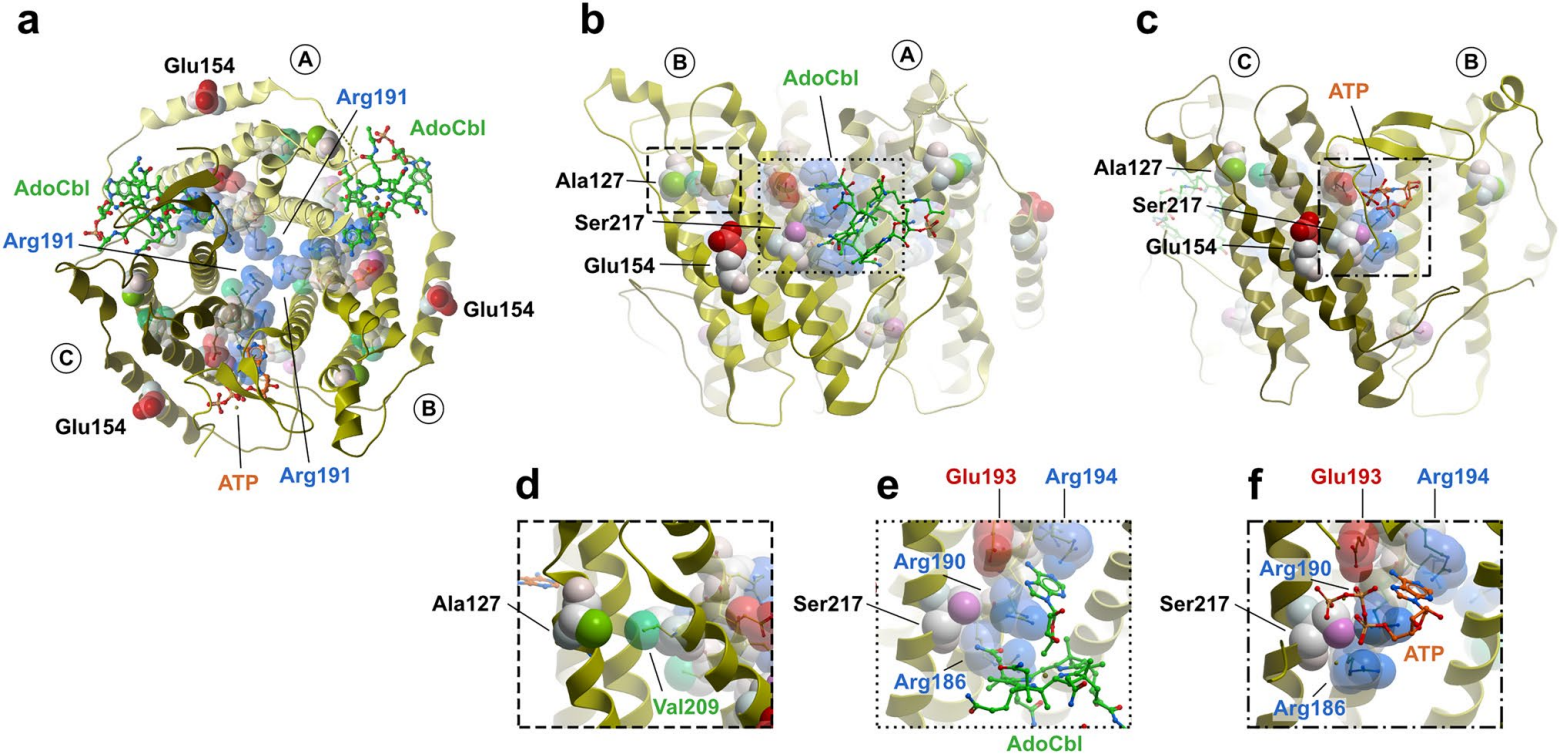
Mapping of pathogenic variants onto the MMAB structure. Sites of frequent and novel missense variants have been depicted onto all three subunits of the MMAB homotrimer (PDB code: 6D5K). (**a**) Top view. (**b**) Side view of a trimeric interface of chains A and B containing AdoCbl. (**c**) Side view of a trimeric interface of chains B and C containing ATP. (**d**) Zoom in from (**b**) to show interaction of Ala127 and Val209. (**e**) Zoom in from (**b**) to show residues involved in AdoCbl binding. (**f**) Zoom in from (**c**) to show residues involved in ATP binding

To get a clearer indication of the potential molecular impact of the novel and frequent missense variants identified in our cohort, we mapped them onto the human MMAB protein structure (Fig. 4). MMAB assembles as a homotrimer with cobalamin and ATP binding sites at the subunit interfaces.

A top down view of MMAB (Fig. 4a), illustrates the positioning of Arg191 in the trimeric core, whereby the frequently identified substitution to tryptophan (p.(Arg191Trp)) would result in a loss of charge and change in side-chain size, which might be expected to result in protein instability. By contrast, Glu154 is a fully solute accessible outer edge residue. The novel identified substitution p.(Glu154Asp) results in a one carbon unit side-chain shortening, but conservation of negative charge. Structurally this appears to be quite a conservative change with no clear consequences, which belies its clinical severity and lack of cobalamin responsiveness (Table 1).

A side view of the MMAB protein, focusing on the AdoCbl (Fig. 4b) and ATP (Fig. 4c) binding pocket at the subunit interfaces, highlights the role of catalytic residues. Just outside of the active site are Ala127 and Val209, which are close enough (4.4 Å) to engage in hydrophobic (van der Waals) interactions (Fig. 4d). We found deleterious variants at both residues in our cohort, including the novel p.(Ala127Asp). These appeared to have a very similar effect on PI activity, and both were responsive to cobalamin supplementation (Table 1). Within the subunit interfaces, residues Arg186 and Arg190, the sites of the frequent deleterious substitutions p.(Arg186Trp/Gln) and p.(Arg190His), along with Glu193 and Arg194, substituted as p.(Glu193Lys) and p.(Arg194Ser) in our cohort, coordinate both AdoCbl and ATP. Ser217, site of the novel variant p.(Ser217Ile), supports this coordination, likely through polar interactions with the side-chain of Arg186 and cobalamin in the presence of AdoCbl (Fig. 4e), and polar interactions with Arg186 and Arg190 in the presence of ATP (Fig. 4f). Substitution to isoleucine at residue 217 would be expected to break these polar interactions, resulting in disruption of the active site. Such an explanation is consistent with the cobalamin non-responsive loss of function identified from an individual homozygous for (p.(Ser217Ile)).

### MMAB biochemistry

To better understand how catalytic variants may affect enzymatic function, we performed a biochemical characterization of human MMAB.

Recombinant human MMAB was expressed as an N-terminally truncated protein, consisting of amino acids 56–250, which omits the mitochondrial leader sequence and incorporates the same residues represented in the recently solved human MMAB structure (Campanello et al. 2018). Final protein purity was > 95% as visualized by SDS-PAGE (Suppl. Figure 2a) with a native molecular weight of 78 kDa (Suppl. Figure 2b), as determined by size exclusion chromatography, corresponding to its biological assembly as a homotrimer (Schubert and Hill 2006; Campanello et al. 2018).

**Fig. 5:**
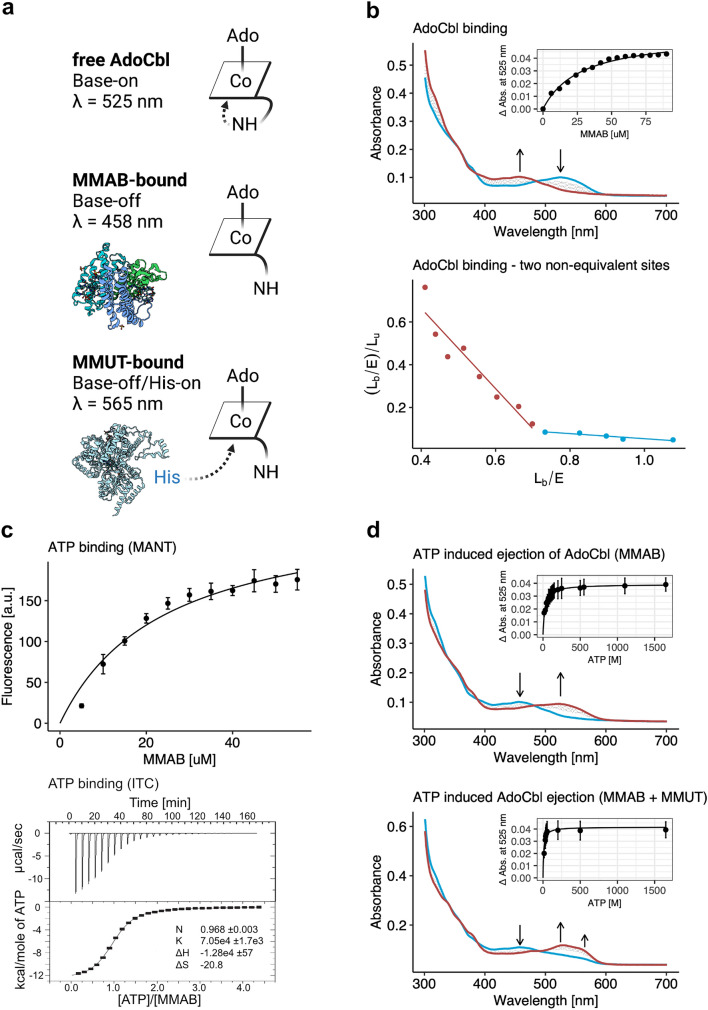
Biochemical characterization of human MMB. (**a**) Schematic representation of different chemical states of AdoCbl and the absorbing wavelengths; modified from (Padovani et al. 2008). (**b**) AdoCbl binding. *Top*. UV–visible absorbance spectrum was obtained by titrating a fixed concentration of AdoCbl with increasing concentrations of MMAB (blue = 0 µM, red = 90 µM). Inset. Change in absorbance at 525 nm. Each data point represents the mean of n = 3. *Bottom.* Scatchard plot analysis of the change in absorbance at 525 nm. Dashed lines represent linear regression fits. (**c**) ATP binding. *Top.* Fluorescence quenching in arbitrary units (a.u.) of MANT-ATP titrated with increasing concentrations of MMAB. Experiment was performed in technical triplicates. *Bottom*. ITC data for binding of ATP to MMAB. Upper panel depicts ATP-binding in power versus time. Lower panel shows integration of data in the upper panel. Since one-binding site was indicated, the data in the lower panel were fit to a single-site binding model. (**d**) ATP-mediated AdoCbl release. *Top*. UV–visible absorbance spectrum changes following titration of ATP (blue = 0 µM, red = 1650 µM) to AdoCbl-bound (holo-)MMAB. Inset. Release of AdoCbl from holo-MMAB represented by change in absorbance at 525 nm. Each data point represents the mean of *n* = 3. *Bottom*. The same as above but performed following pre-incubation of holo-MMAB with apo-MMUT; AdoCbl binding to MMUT is indicated by a transition at 565 nm

As a first characterization, we examined binding to AdoCbl using spectrophotometry. Titration of increasing concentrations of MMAB to AdoCbl revealed a transition from an absorption maximum at 525 nm, consistent with the “base-on” six-coordinate state of free AdoCbl, to an absorption maximum at 458 nm (Fig. 5a, b top), corresponding to the “base-off” five-coordinate state of MMAB-bound AdoCbl (Padovani et al. 2008). Curve fitting of the change in absorbance at 525 nm to a one-site binding model suggests a provisional dissociation constant (*K*_d_) = 27.6 ± 2.0 µM (Fig. 5b top inset). However, Scatchard analysis indicates the presence of two non-equivalent binding sites, with *K*_d1_ = 0.55 μM and *K*_d2_ = 8.4 μM (Fig. 5b bottom). This latter analysis is more consistent with values published for the bacterial homologue ATR (*K*_d1_ = 0.14 ± 0.02, *K*_d2_ = 2.1 ± 0.5 μM, (Padovani and Banerjee 2009) and the recently published *K*_d_ = 0.96 ± 0.31 for MMAB (Campanello et al. 2018).

We next examined binding of ATP to apo-MMAB in two ways. First, we used spectroscopy of the fluorescent nucleotide analogue 2',3'-O-(N-Methyl-anthraniloyl)-adenosine-5'-triphosphate (MANT-ATP). Using titration of MMAB to MANT-ATP and monitoring the resulting change in fluorescence yielded a *K*_d_ = 21.1 ± 3.4 µM (Fig. 5c top). Binding specificity was confirmed by competitive exchange of MANT-ATP with unlabelled ATP (Suppl. Figure 3a). Second, as an orthogonal assay, we determined ATP binding by isothermal titration calorimetry. Following injection of ATP to MMAB we identified a *K*_d_ = 14.2 ± 0.6 µM (Fig. 5c bottom), comparable to that of MANT-ATP. The isothermal calorimetry measurement further identified an *N* = 0.968 ± 0.0032, indicating that the three potential ATP binding sites have equal affinity, while a negative ΔH suggested that binding is enthalpically driven.

Release of AdoCbl from MMAB can be initiated by binding to ATP (Padovani and Banerjee 2009). We monitored this release spectrophotometrically, by measuring the dose-dependent increase in absorption at 525 nm and decrease at 458 nm following addition of ATP to AdoCbl-bound MMAB (holo-MMAB) (Fig. 5d top). Curve fitting of the change in absorbance at 525 nm indicates ATP-induced released of AdoCbl has an activation constant (*K*_a_) = 23.9 ± 4.8 µM (Fig. 5d top inset). This *K*_a_ is in line with the *K*_d_ of apo-MMAB for ATP (Fig. 5c), suggesting the affinity of MMAB for ATP is not significantly impacted by the presence of bound AdoCbl. Since the absorbance spectrum generated after AdoCbl release by ATP is comparable to the absorbance spectrum generated by free AdoCbl, we expect that the cofactor was fully released into solution.

### Altered MMAB biochemistry in the presence of MMUT

We previously found that release of AdoCbl from MMAB upon ATP binding is favored in the presence of MMUT (Plessl et al. 2017). Here, we pre-incubated holo-MMAB with purified recombinant human MMUT (Suppl. Figure 2a), which retains its biological assembly as a dimer (Suppl. Figure 2b). Consistent with our previous findings, addition of ATP in the presence of MMUT resulted in a *K*_a_ of 13.3 ± 1.3 µM (Fig. 5d bottom inset), which is reduced compared to MMAB alone. Here, released AdoCbl was not free in solution, but instead bound by MMUT, as indicated by the additional absorbance peak at 565 nm (Fig. 5d bottom, Suppl. Figure 3b). We did not identify cofactor transfer from MMUT pre-loaded with AdoCbl (holo-MMUT) to MMAB (Suppl. Figure 3c). The direct binding of AdoCbl by MMUT, as well as the reduced *K*_a_ for ATP by MMAB in the presence of MMUT, suggests a direct transfer of AdoCbl from MMAB to MMUT, in line with findings from bacterial homologs (Padovani et al. 2008).

## Discussion

In this study, we extended the landscape of known pathogenic *MMAB* variants and relate them to crucial biochemical and clinical parameters. Sequencing of 97 individuals confirmed bi-allelic variants in the *MMAB* gene. In line with previous work (Lerner-Ellis et al. 2006), we found most pathogenic missense variants to form a hotspot cluster at the end of exon 7. Within this cluster, the most frequently identified variant was p.(Arg186Trp). This substitution has been found to be associated with early-onset disease (Lerner-Ellis et al. 2006), consistent with our data. Structurally, Arg186 is situated at the subunit interface involved in AdoCbl and ATP binding, and its substitution to tryptophan would be predicted to disrupt their binding as well as proper protein folding, both of which have been shown experimentally (Zhang et al. 2009). The latter pathomechanism (misfolding) has also been found for the second most frequently identified missense variant in our cohort p.(Arg191Trp) (Jorge‐Finnigan et al. 2010), an outcome structurally predicted due to the placement of Arg191 in the trimeric core.

The most frequently identified truncating variant was p.(Gln234*). It and p.(Tyr219Serfs*4) are found in exon 9, which encodes the C-terminus of MMAB. Due to their position at the 3′ of the coding sequence, it appears likely that alleles harboring these variants escape nonsense-mediated decay and encode for partially functioning proteins. This explanation is congruent with our in vitro findings, and those of others (Lerner-Ellis et al. 2006), that indicate fibroblasts carrying p.(Gln234*) on at least one allele show consistent in vitro responsiveness to OHCbl supplementation. In this study, we extend this knowledge by demonstrating that affected individuals are also clinically responsive to vitamin B_12_ treatment. Contrary to previous reports (Lerner-Ellis et al. 2006), individuals harboring this change on one or two alleles in our cohort showed a tendency to present with a later disease onset, after the neonatal period. Biochemical interrogation of this truncation using a bacterial (*M. extorquens*) homolog of MMAB suggested this C-terminal loss has only modest effects on enzymatic activity, but influences transfer of AdoCbl to MMUT (Lofgren and Banerjee 2011). Such a mechanism is consistent with our findings and would be interesting to examine further.

In vitro examination of OHCbl responsiveness is an established diagnostic instrument for MMA. As applied to individuals with MMUT deficiency (*mut*-type MMA), a PI ratio above 1.5 (*mut*^−^ subtype) has been associated with a milder clinical phenotype than a PI ratio below 1.5 (*mut*^0^ subtype) (Hörster et al. 2007; Forny et al. 2016). Nevertheless, to our knowledge in vitro and clinical responsiveness in *mut*^−^ and *mut*^0^ individuals have not been directly compared. Here, we used the same PI ratio cut-off to delineate cobalamin responsiveness in MMAB deficiency (*cblB*-type MMA). For the individuals with available data and a PI ratio above 1.5 we found that they have lower plasma ammonia and a later disease onset, two parameters representative of a milder clinical phenotype, similar to MMUT deficiency. Therefore, a PI ratio cut-off of 1.5 seems useful and applicable in *cblB*-type MMA as well and may even warrant corresponding subtype classification into *cblB*^−^ and *cblB*^0^. Importantly, we have even gone a step further and shown that in our cohort, in vitro responsiveness is indeed associated with clinical responsiveness. This may have practical application, as it truly sets the stage for rigorous clinical testing of cobalamin responsiveness in *cblB*-type MMA, which now could be guided by in vitro results.

To discern pathomechanisms of MMAB deficiency, it is important to understand how the protein functions at the biochemical level. Until now, most of our knowledge in this area has been derived from bacterial protein homologs. From this study, we can help put those findings into the context of the human enzyme. Although MMAB has three potential active sites, we found AdoCbl to bind at two non-equivalent sites, each with low micromolar affinity. This is consistent with a bacterial homolog from *M. extorquens*, which was shown to bind AdoCbl at two non-equivalent sites (*K*_d1_ = 0.14 μM; *K*_d2_ = 2.1 μM) (Padovani and Banerjee 2009). Interestingly, it is in contrast to a recent examination of MMAB, which identified only a single equivalent site (*K*_d1_ = 0.96 μM) (Campanello et al. 2018). Further, we found a MMAB to bind ATP with equivalent affinity at all active-sites, having a dissociation constant of approximately 20 µM for the fluorescent ATP derivative MANT-ATP, and 14 µM for ATP itself, the latter assessed by isothermal calorimetry. Examination of MANT-ATP binding to the protein homolog from *M. extorquens* revealed K_d_’s of 0.19 and 5.9 µM, while isothermal calorimetry suggested two ATP molecules bind per trimer (*K*_d1_ = 0.60 µM; *K*_d2_ = 12.6 µM) (Padovani and Banerjee 2009)*.* A recent examination of MMAB by the same group also suggested a high (*K*_d1_ = 6.3 µM) and a low (*K*_d2_ = 134 µM) affinity site (Campanello et al. 2018). While our findings are more consistent with the MMAB experiments, it is yet unclear why they found inequivalent high and low affinity sites while we identified only one site with intermediate affinity by both methods. The subtle differences in AdoCbl and ATP binding between bacterial and human proteins appear to result in an important difference in ATP-mediated AdoCbl release. Stop-flow experiments performed with bacterial enzyme identified only one molecule of AdoCbl to be released after ATP addition, which required near millimolar concentrations (*K*_a_ = 870 µM) (Padovani and Banerjee 2009). Our experiments suggest AdoCbl is fully released from both sites in MMAB after addition of ATP, which takes place at a concentration > 35 times lower (*K*_a_ = 24 µM). Although both bacterial and human proteins are sensitized to AdoCbl release by the presence of the cofactor utilizing enzyme (in humans, MMUT), the gulf in required ATP concentrations to activate this release remains (bacterial *K*_a_ = 250 µM; MMAB *K*_a_ = 13 µM) (Padovani and Banerjee 2009). Thus, although the overall function and architecture of MMAB is conserved from bacteria, subtle changes in affinities have resulted in large changes to activity, which in turn is likely to play a direct role in how variations lead to pathogenicity.

In conclusion, we have examined a cohort of 97 patients with *cblB*-type MMA, providing evidence of novel variants, confirmation of frequent variants, and supportive proof that in vitro functional analysis can be translatable to clinical effect. This, along with our biochemical characterization, expands the knowledge of the molecular basis of this disease, and facilitates insight into MMAB function.

## Supplementary Information

Below is the link to the electronic supplementary material.

Supplementary file1 (PDF 1007 KB)
