# Supplementary material for: Spectrum and characterization of bi-allelic variants in MMAB causing cblB-type methylmalonic aciduria
Source: Hum Genet. 2021 Nov 18;141(7):1253–67. doi: 10.1007/s00439-021-02398-6 (PMC9262797; doi:10.1007/s00439-021-02398-6)
Supplement: Supplementary file 1 — Supplementary file1 (PDF 1007 KB) [file 439_2021_2398_MOESM1_ESM.pdf]

Spectrum and characterization of bi-allelic variants in *MMAB* causing *cblB*-type methylmalonic aciduria

Patrick Forny[1], Tanja Plessl[1], Caroline Frei[1], Celine Bürer[1], D. Sean Froese[1], Matthias R. Baumgartner[1]

[1] Division of Metabolism and Children's Research Center, University Children's Hospital Zurich, University of Zurich, Zurich, Switzerland

Supplementary Information

Supplementary Figure 1

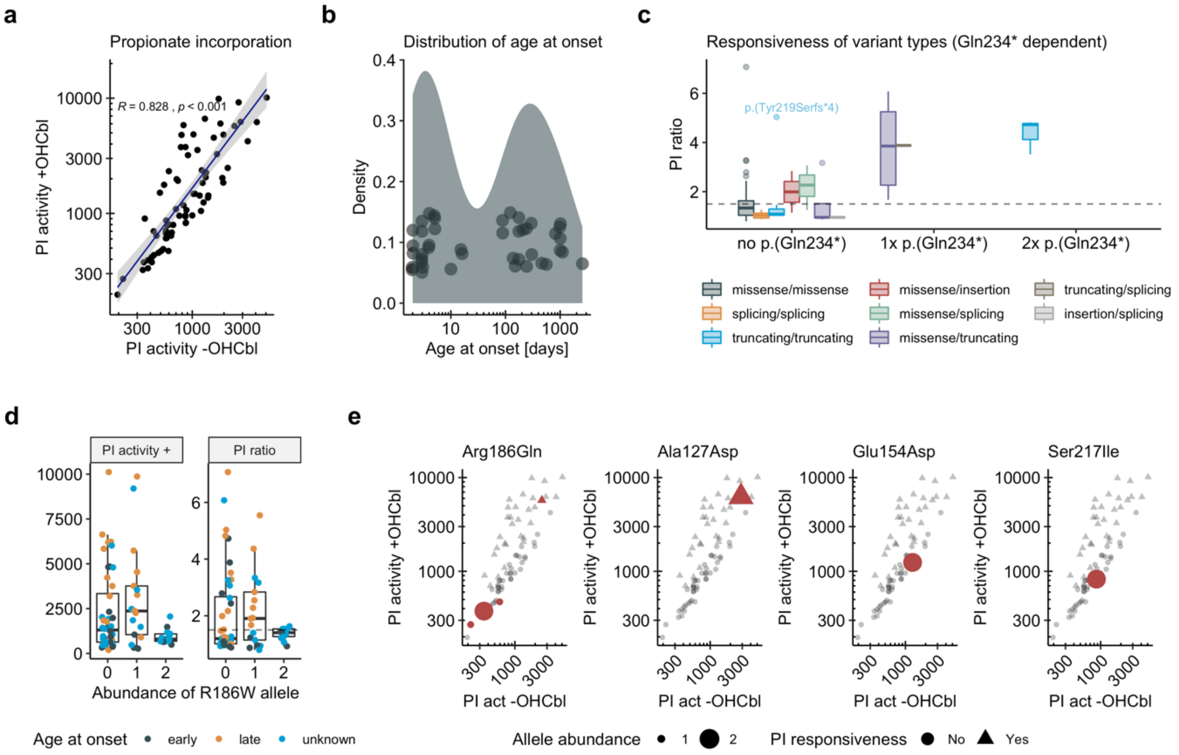

Suppl. Fig. 1. (**a**) Scatter plot of PI activity with and without supplementation of OHCbl; linear regression calculated by Pearson correlation. (**b**) Density distribution plot of the age of onset of patients with available information; dots represent individual patients; y axis jitter was added for better visibility. (**c**) PI ratios according to allele combinations, grouped based on the abundance of the p.(Gln234*) allele. (**d**) PI activity supplemented with OHCbl and PI ratio of all samples, grouped based on the abundance of the p.(Arg186Trp) allele. (**e**) Scatter plot of PI activity with and without supplementation of OHCbl; specific alleles (plot title) are highlighted in red; triangles indicate in vitro responsiveness, dot or triangle size indicate the abundance of the specific allele (plot title).

Supplementary Figure 2

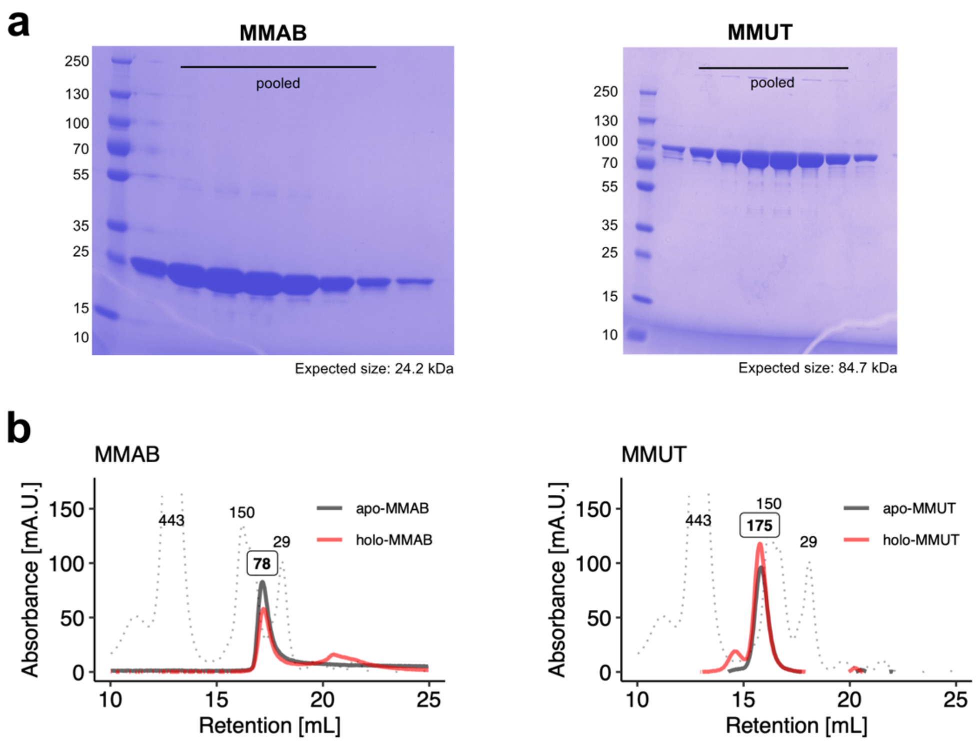

Suppl. Fig. 2. (**a**) SDS-PAGE gels indicating high purity (>95%) after protein purification for MMAB, MMUT, and MMAA. (**b**) Size-exclusion chromatograms with indicated peaks for the respective proteins (MMAB as homotrimer, MMUT as homodimer); dotted curve represents a standard sample allowing calculation of protein size.

Supplementary Figure 3

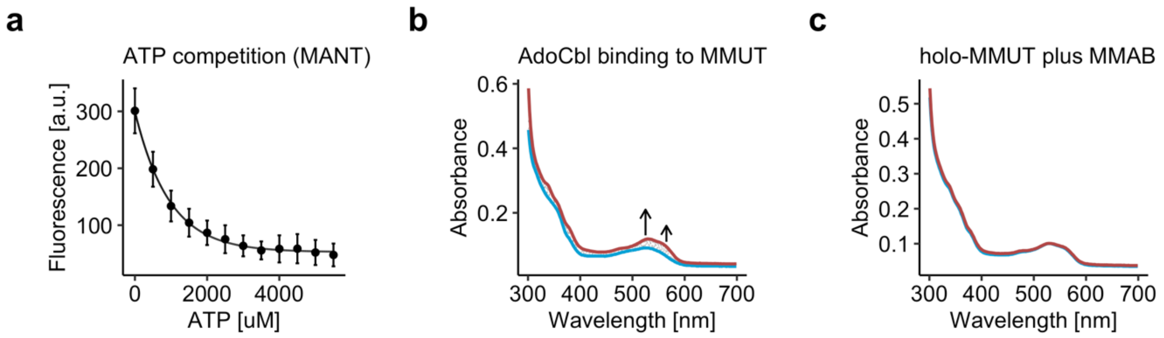

Suppl. Fig. 3. (**a**) Increasing ATP concentrations lead to exchange for MANT-ATP at the binding site of MMAB. (**b**) Absorbance spectrum of AdoCbl under increasing concentrations of MMUT (blue line, 0 μM of MMUT; red line, 80 μM of MMUT added). (**c**) Increasing concentration of MMAB [0-60 μM] were added to holo-MMUT, revealing no change of the absorbance spectrum.

Supplementary Table 1

Exon	Direction	Oligonucleotide sequence	PCR product size (bp)
1	Forward	ACC AAT AAA AAT TGA AAC CTG AGC G	365
1	Reverse	AAC AGC GTG GAG ACG TCA CCT GAC	
2	Forward	AGA TGA CCA TCT AAG CAG TAA GG	304
2	Reverse	CGC GCC TGG TAT ATT ATA CTT TA	
3	Forward	GGA GGT CAA GAC TAG CTC GG	521
3	Reverse	CAA TAA GGG CTG ACA ACC	
4	Forward	GAC TGG AAA TAA TGA GCA TCC GA	357
4	Reverse	TAA CTG GTG GCT GGA TGC TGA GT	
5	Forward	CAC CTC CAA CTC TGG GGA TCA CA	339
5	Reverse	TGG GAT CCC AGA TGG TGA CCC TA	
6	Forward	CAC GGG TGG GTC AGT TAG CCT TGC T	386
6	Reverse	TTG GGG AAC TGG AGG ACA GCG TCT	
7	Forward	ATC CCT CTT TTC ACA GTG ACA GA	483
7	Reverse	GTC TGT TGA TGT GTG GCT GGA TG	
8	Forward	CCA GGT GGC TCC CTC AAC TCA	563
8	Reverse	CAG CAG CCT CTC CCA ATA AGA	
9	Forward	GCA GAA GAG CTG ACA GCG TA	1101
9	Reverse	GGT GGC AAG AGG TGT AGA TCA	

Suppl. Table 1. Sequences of primers used in Sanger sequencing.
